# Letter to the editor regarding the editorial “Quo vadis? – Medical education 2020 between politics and science”

**DOI:** 10.3205/zma001038

**Published:** 2016-05-17

**Authors:** Jean-François Chenot, Jost Steinhäuser, Antje Bergmann, Maren Ehrhardt, Johannes Spanke, Anne Simmenroth

**Affiliations:** 1 German College of General Practitioners and Family Physicians (DEGAM), Spokesmen of the Section Postgraduate Medical Education, Berlin, Germany; 2 University Medicine Greifswald, Department of General Practice, Greifswald, Germany; 3 University Medical Centre Schleswig-Holstein, Campus Lübeck, Institute of General Practice, Lübeck, Germany; 4 German College of General Practitioners and Family Physicians (DEGAM), Spokeswomen of the Section Undergraduate Medical Education, Berlin, Germany; 5 University Medical Centre Carl Gustav Carus of the Technical University Dresden, Section General Practice, Dresden, Germany; 6 University Medical Centre Hamburg-Eppendorf, Institute of General Practice, Hamburg, Germany; 7 University Medicine Göttingen, Institute of General Practice, Göttingen, Germany

## Letter to the editor

Harendza et al. are criticizing the politicisation and lack of scientific underpinning for current reform of undergraduate medical education [1]. Their analysis has „blind spot“ regarding the relation of undergraduate and postgraduate medical education. Unlike in many other countries in German speaking countries postgraduate medical education is neither linked nor mandatorily attached to university medical centres. Undergraduate medical education is financed by the government which regulates the content through the Licensing Regulations for Physicians *(Approbationsordnung)*. Graduate medical education is regulated by the state medical associations and provided mainly by hospitals. Often the need of getting the work done is granted a higher priority than need for supervised learning. Changing the Licensing Regulations for Physicians is only way the federal Government can address deficits in patient care and physician’s qualifications, e.g. care for the elderly, pain management or palliative care. This explains the recent introduction of new interdisciplinary teaching modules and the strengthening of Primary Care/General Practice in undergraduate medical education. However postgraduate medical education might be more appropriate to foster specific clinical skills at higher level of competence. So far postgraduate medical education consists mostly of “learning on the job” without structured rotation or theoretical underpinning. While didactic qualifications are more and more important in undergraduate teaching this is completely neglected in postgraduate training. The National Competence Based Catalogues of Learning [http://www.nklm.de] attempted to create a link between undergraduate and graduate medical education. Due to lack of structure of postgraduate medical education and since the regulations for postgraduate medical training are not a curriculum, this was doomed to fail.

The expressed fear of an overdose of General Practice in medical school is unwarranted, it is rather the overdoses of medicine at all, if every subspecialty attempts presenting itself in full width to medical students. More General Practice is more likely part of the solution and could help to focus on essentials thus creating the freedom for self-determined learning. The unbalanced dominance of hospital care in undergraduate medical education is comprehensible from a historical perspective but cannot reasonably be defended; given that most patients are being cared for in the ambulatory setting and roughly half of the physician work force is in ambulatory care. Again a common problem of under- and postgraduate medical education, where due to the lack of financing most training is completed in a hospital setting. Sufficient competence in preventive services or long term care for chronic conditions cannot be acquired there. Strengthening General Practice, by introducing a mandatory final year rotation is an opportunity, particularly for medical students striving for specialist training, to get a better insight in the interface between ambulatory and hospital care. This is therefore rather an epidemiologically and didactically warranted step than a mere political decision. Exposure to a specialty and rural experience determine if working in rural areas is taken into consideration at all [2], [3]. Persuading medical students to work as General Practitioner in rural areas is certainly not the task of academic General Practice. The most important factor for rural practise as generalists or specialists is being brought up rural. This could easily be taken into account when selecting medical students.

## Competing interests

The authors declare that they have no competing interests.

## References

[1] Harendza S, Fischer MR, Fabry G (2016). Quo vadis? - Medizinstudium 2020 zwischen Politik und Wissenschaft. GMS J Med Educ.

[2] Schneider A, Karsch-Völk M, Rupp A, Fischer MR, Drexler H, Schelling J, Berberat P (2013). Determinanten für eine hausärztliche Berufswahl unter Studierenden der Medizin: Eine Umfrage an drei bayerischen Medizinischen Fakultäten. GMS Z Med Ausbild.

[3] Steinhäuser J, Joos S, Szecsenyi J, Götz K (2013). Welche Faktoren fördern die Vorstellung sich im ländlichen Raum niederzulassen?. Z Allg Med.

